# Hormesis in Aging and Neurodegeneration—A Prodigy Awaiting Dissection

**DOI:** 10.3390/ijms140713109

**Published:** 2013-06-25

**Authors:** Lei Mao, Jacqueline Franke

**Affiliations:** 1 Department of Life Science Engineering, HTW Berlin, University of Applied Sciences, Wilhelminenhofstraße 75A, Berlin 12459, Germany; E-Mail: jacqueline.franke@htw-berlin.de; 2 Institute of Medical Genetics and Human Genetics, Charité—Universitätsmedizin Berlin, Augustenbruger Platz 1, Berlin 13353, Germany

**Keywords:** hormesis, aging, neurodegenerative diseases (ND), reactive oxygen species (ROS), reactive nitrogen species (RNS), mathematical modeling, personalized medicine

## Abstract

Hormesis describes the drug action of low dose stimulation and high dose inhibition. The hormesis phenomenon has been observed in a wide range of biological systems. Although known in its descriptive context, the underlying mode-of-action of hormesis is largely unexplored. Recently, the hormesis concept has been receiving increasing attention in the field of aging research. It has been proposed that within a certain concentration window, reactive oxygen species (ROS) or reactive nitrogen species (RNS) could act as major mediators of anti-aging and neuroprotective processes. Such hormetic phenomena could have potential therapeutic applications, if properly employed. Here, we review the current theories of hormetic phenomena in regard to aging and neurodegeneration, with the focus on its underlying mechanism. Facilitated by a simple mathematical model, we show for the first time that ROS-mediated hormesis can be explained by the addition of different biomolecular reactions including oxidative damage, MAPK signaling and autophagy stimulation. Due to their divergent scales, the optimal hormetic window is sensitive to each kinetic parameter, which may vary between individuals. Therefore, therapeutic utilization of hormesis requires quantitative characterizations in order to access the optimal hormetic window for each individual. This calls for a personalized medicine approach for a longer human healthspan.

## 1. Introduction

Since ancient times, people have proposed (and practiced) the idea that low doses of poison could be beneficial to health in the long run. The German philosopher Friedrich Nietzsche (1844–1900) made the famous statement that “What doesn’t kill us makes us stronger” (*Was uns nicht umbringt macht uns stärker*), probably to excuse his drinking habit. The old German Kanzler, Helmut Schmidt (born 1918), is well-known for his habitual cigarette consumption, however, even as a nonagenarian, he is admired both for his sharp thinking, and for his political engagement.

Indeed, studies have shown that dietary intake of moderate amounts of ethanol can double the lifespan of nematodes [1] and enhance memory in mice [2]. In humans, it is well-known that high ethanol consumption is associated with heart disease, liver cirrhosis, neurological disorders and cancer [3]. However, moderate drinkers have overall reduced mortality, especially of the kinds resulting from coronary heart disease and stroke [3,4]. In a similar vein, the apparent protective effect of cigarette smoking on Parkinson’s disease (PD) is one of the few consistent results in epidemiology [5,6], and could have a similar effect on Alzheimer’s dementia [7].

**Figure 1:**
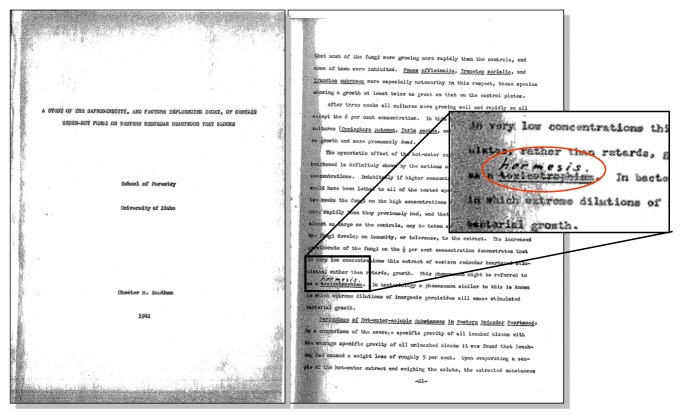
The first appearance of the term “hormesis” can be traced back to 1941 in the undergraduate thesis of Chester Southam as a better substitution for the word “toxicotrophism”. There, he reported that low doses of the toxic red cedar tree extract enhanced the proliferation of fungi with an overall biphasic shape of the dose response (credit: http://www.dose-response.org).

Such phenomena, distinguished by beneficial effects of apparently toxic agents at their low doses, have been known for centuries. Back in 1888, the German pharmacologist Hugo Schulz observed that small doses of multiple chemical disinfectants stimulate yeast growth [8,9]. However, the true origin of the word “hormesis” (*derived from the ancient Greek term hormáein, which literally means “to excite”, or “to set in motion”*) dates back to Chester Southam’s undergraduate thesis in 1941 as a better substitution for the word “*toxicotrophism*” (Figure 1). According to the original publication of Southam and Ehrlich in 1943, “*The term hormesis (adj. hormetic) is proposed to designate such a stimulatory effect of sub-inhibitory concentrations of any toxic substance of any organism*.” [10].

Essentially describing an overall biphasic shape of the dose response curve, hormesis seems to be quite commonplace: Bacteria tend to flourish in the presence of tiny amounts of antibiotics [11] and nematodes that have experienced a variety of environmental stresses including heat, hypoxia or elevated reactive oxygen species (ROS) exhibit extended lifespan [12]. Insects treated with low doses of pesticides or ethanol generally live longer and produce more eggs [11]. Short term ischemic treatment of human tissues increases their resistance to subsequent, long-term ischemia-reperfusion damages [13]. Small doses of even the most harmful substances such as dioxin can stimulate beneficial responses that enhance the organism’s resistance against multiple stresses [14]. With controversial, hormesis effects have also been observed with low dose radiation [15,16]. Recently, the term hormesis has been borrowed by the field of neurodegenerative diseases (ND) to describe possible protective effects of some neurotoxins. For example, amyloid-beta protein (Aβ) and the development of amyloid plaques have been considered as a hallmark in Alzheimer’s disease pathology [17]. However, at physiological concentration, Aβ may actually have a protective effect on neurons [18]. It enhances long term potentiation and memory, increases neurite outgrowth and produces presynaptic enhancement of neuronal plasticity [19].

Despite all these common examples, hormesis has been a topic of vigorous debate since its introduction [20,21]. Homeopathic practitioners worship the hormetic concept as the ultimate support for the therapeutic usage of highly poisonous drugs, whereas nonbelievers fiercely refuse this concept due to its principle reversal of toxicological dogma [21].

It is to be noticed here that, objectively, the term hormesis *per se* is merely a phenomenological description. In other words, hormesis is a symptom, not a diagnosis. Just like fever can have multiple medical diagnoses, the underlying mechanism of hormetic effects could be largely heterogeneous. Thus, a particular hormetic activity can only be fully described if there is an understanding of the biological processes underpinning that specific biphasic dose response [21]. Edward Calabrese, an enduring advocate of the hormesis concept proposed the theory that low doses of toxins may trigger certain defensive responses that enable the organism to become more resistant to the same or similar stimuli [22]. Indeed, an increasing number of hormetic effects have already been assigned to clear mechanisms: As a classical example, thermal stress induces the gene expression of heat-shock proteins [23,24]. The exposure to low dose pesticides can induce xenobiotic detoxification enzymes such as cytochrome P450 [25]. Low-dose ultra violet (UV) radiation enhances the DNA base excision repair enzyme [26]. In a sense, the development of acquired immunity through vaccination can also be termed hormesis, with its well-known pathogen T-cell interactions [27].

During the last decade, the word hormesis as the principle behavior of the stress-response has found a new lease on life in an old context [20]: the biology of aging and age-related diseases [28]. One reason for this is that hormetic effects typically occur over a relatively long time scale. Importantly, however, as mechanisms of aging have been revealed more and more clearly, people foresee potential, but immense, advantages of hormetic effects on aging [29,30]. Built on current understandings, this review aims to discuss the effects of hormesis in the context of aging and neurodegenerative diseases, with a special focus on the underlying hormetic mode-of-action involving reactive oxygen species. We will also envision how future studies will continue to refine our knowledge in this field, ultimately allowing us to profit from hormetic medical approaches.

## 2. ROS Hormesis in Aging and Neurodegenerative Diseases

Aging can be thought of as the multi-causal progressive failure of organism maintenance [30], with death as the final manifestation of the breakdown in homeostasis [31]. Danham Harman’s free radical theory is one of the major hypotheses on aging mechanisms. It states that reactive oxygen species induce stochastic occurrence and accumulation of macromolecular damages, leading to a progressive decrease in the organism’s molecular fidelity [31]. As the major biological building block, proteins are one of the prime targets for oxidative damage. The negatively charged superoxide anion (O_2_^•−^) targets iron-sulfur clusters in many proteins. Neutrally charged hydrogen peroxide (H_2_O_2_) reacts preferably with the cysteine thiols, leading to the generation of reversible and irreversible protein carbonyl derivatives. Oxidative damage also affects DNA and lipids, resulting in the accumulation of partially irreversible damages such as 8-oxo-7,8-dihydroguanine or peroxidized lipids [32,33].

A considerable portion of intracellular ROS results from the energy-producing metabolic activities of mitochondria. It is estimated that about 2% of oxygen consumption is converted to ROS during oxidative phosphorylation [34]. Auto-oxidation of reduced respiratory components of the mitochondrial electron transport chain causes production of the superoxide anion and hydrogen peroxide. In the presence of iron, these can produce the highly reactive hydroxyl radical OH· via the Fenton reaction. OH· radicals can initiate chain reactions of lipid peroxidation while generating peroxyl- and alkoxyl radical intermediates [35]. Moreover, mitochondrial superoxides may react with nitric oxide to produce peroxynitrite (ONOO^−^), a strong oxidant that can cause overwhelming oxidative injuries [36] (though reactive nitrogen species (RNS) are not the focus of this review).

Being both a major source, as well as the primary target of potentially harmful ROS, mitochondrial dysfunction is strongly associated with the onset of numerous age-related diseases [37], such as diabetes [38], cancer [39] and neurodegenerative conditions including Parkinson’s disease and Alzheimer’s disease [40,41]. Indeed, impairment of mitochondria has been assumed to be a main driving force of aging in itself [42,43].

### 2.1. ROS and Aging: Causal but Not Concomitant

Plenty of experimental findings have demonstrated increasing levels of ROS during organism aging [35]. However, across aging models from yeast to mouse, enhanced ROS detoxification via endogenous or exogenous antioxidants failed to show consistent effects on lifespan [44]. For instance, neither global reduction nor over-expression of manganese superoxide dismutase (SOD2), a major scavenger of superoxide molecules, alters lifespan, even though the enzyme activity was high enough to counteract the strong ROS effect of paraquat (*N*,*N*′-dimethyl-4,4′-bipyridinium dichloride) [45,46].

Moreover, numerous clinical interventions were unable to establish a positive association between antioxidant supplements and benefits to health: Most studies found a lack of correlation, and several studies even suggested severe adverse effects of antioxidant supplements on aging retardation [47,48]. Interestingly, this is consistently the case when antioxidants were applied simultaneously with lifespan-promoting measurements such as diet restriction or physical exercise [49–51]. For instance, it has been shown that long-term ascorbic acid supplements, an efficient free radical scavenger, diminished the exercise-induced adaptive responses in humans [52–54]. Combined, these facts raise the hypothesis that ROS may act as essential mediators promoting health and longevity in the frame of certain physiological adaptation processes [55,56].

### 2.2. Caloric Restriction Induced Lifespan Extension Is Mediated by ROS

As currently the most robust lifespan modulating measure; caloric restriction (CR); a 20%–40% reduction in caloric intake without malnutrition; has been consistently shown to increase lifespan in almost all model organisms investigated [28,57]. Rhesus monkeys under long-term CR demonstrated a prolonged time period of healthy living (or a prolonged “healthspan”); although the effect of CR on lifespan extension remains controversial [58]. Profound beneficial effects of CR on health have also been frequently reported. For example; CR in humans clearly reduces the risk of type 2 diabetes and cardiovascular disease [59,60]. Long-term CR was reported to be highly effective in reducing the risk of atherosclerosis [61]; accompanied by significant improvement of vascular functions [62]. Neuroprotective effects and promotion of adult neurogenesis were also observed in CR rodents [63].

For a period of time; the idea that CR exerts its beneficial effects by reducing the level of ROS; which is associated with a presumably decreased metabolic rate; was seen as a logical extension of Harman’s theory. However; accumulating evidence rejects this common expectation. It is now affirmed that CR increases the organism’s bodyweight-specific metabolic rate in all aging models investigated. This is observed through increased oxygen consumption; increased heat production; as well as increased total energy expenditure as a function of body mass [64–66]. It is probable that the initial energy deficit as a consequence of CR leads to an extensive aerobic metabolic shift in order to counteract it; this phenomenon was termed “mitohormesis” [67]. Increased oxidative phosphorylation under CR has been observed in yeast; nematodes [68–70]; and in man [71]. In addition; it has been demonstrated that CR also promotes mitochondrial biogenesis via a nitric oxide-mediated mechanism [72,73]. Importantly; such enhanced mitochondrial respiration is indeed correlated with an increased oxidative stress level [74]; as exemplified by an increased hydrogen peroxide concentration [75]. Similarly, ROS up-regulation was also connected to the lifespan extension and health benefits associated with physical exercise in humans [35,76,77].

In 2007, it was reported that lifespan extension by CR in nematodes does require ROS [69]. This was demonstrated by an elegant experiment employing two drugs that generate ROS: the respiratory inhibitor azide, which inhibits the respiratory complex IV and promotes H_2_O_2_ generation; and paraquat, which undergoes redox cycling to produce superoxides *in vivo*. At certain dosages, both of these ROS-inducers led to CR-like lifespan extension. Intriguingly, this effect was clearly abolished by the presence of antioxidants [69], and additional evidence was subsequently provided by other researchers as well [67,78]. These findings provided solid proof that an increased ROS formation, at least transiently, is essential for these promotions in longevity [33,69]. Bearing ROS’ damaging potential in mind, it becomes apparent that ROS can be justified as hormetic agents.

### 2.3. The Neuroprotective Potential of ROS Hormesis

Apart from the lifespan extending effect, it has been shown that ROS hormesis can also exert protective effects against neurodegenerative conditions [79]. For example, Bonilla-Ramirez and co-workers treated the *parkin* knock-down *Drosophila melanogaster* with ROS-inducers such as paraquat and polyphenols (*i.e.*, quercetin). Under these conditions, significantly higher lifespan as well as improved locomotor activity was observed in comparison to untreated controls [80].

Accumulating evidence of the possible neuroprotective potential of ROS hormesis comes from studies on a spectrum of substances called CR-mimetics, *i.e.*, drugs or endogenous proteins that mimic some beneficial effect of CR. It was shown that low doses of resveratrol (4 μg/mouse/day) stimulated sensory neurons, and promoted hippocampal neurogenesis in the *dentate gyrus* and subventricular zone of adult mice [81]. Within this hormetic window, cytokines such as BDNF (brain-derived neurotrophic factor) were released from the resveratrol-treated cells, resulting in both autocrine and paracrine neuroprotection. Interestingly, in a parallel study, high doses of resveratrol were shown to significantly inhibit proliferation and survival of mouse neuroprecursor cells both *in vitro* (>10 μM) and *in vivo* (25–250 μg/mouse/day) [82]; this is proposed to function via a ROS-mediated inhibition of BDNF secretion [83,84]. Together, these studies clearly demonstrated a biphasic hormetic curve of the neuroprotective effect of resveratrol, which is possibly mediated by ROS [85,86]. A similar mode-of-action could be assigned to curcumin, olive oil or green tea catechins [87,88]. Moreover, it has recently been shown that over-expression of neuroglobin, an oxygen binding and peroxynitrite-generating protein of the brain, can prevent or limit neuronal damage from beta-amyloid-induced neurotoxicity, stroke, and seizures in mice [89,90].

Apparently, the endogenously produced ROS stimulate certain adaptive responses that protect the organism from damages beyond ROS [46,56,57]. Hence, the logical next step is to depict the actual molecular mechanisms of these downstream adaptive responses.

## 3. ROS-Mediated Adaptive Responses

**Figure 2:**
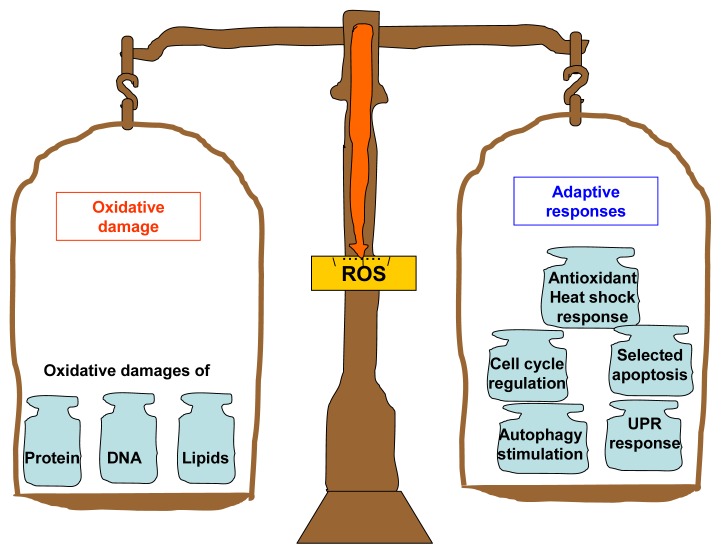
Summary of the bifurcated effects that can be induced by ROS. On the one hand, ROS induces the oxidative damage to proteins, DNA and lipids. On the other hand, they also trigger the organism’s adaptive responses including antioxidant and heat shock responses, fatty acid deacylation-reacylation, cell cycle regulation, DNA repair and apoptosis, unfolded protein responses, and autophagy stimulation. On the other hand, they also trigger the organism’s adaptive responses including antioxidant and heat shock responses, cell cycle regulation and apoptosis, unfolded protein responses, and autophagy stimulation.

Almost six decades after the birth of Harman’s free radical theory, we now know that ROS and RNS are essential in many physiological processes [35,91]. In effect, although some of these species are indeed free radicals (paramagnetic molecular species with an unpaired electron), not all of them are equally reactive. Some of the species, such as superoxides, hydrogen peroxide or nitric oxide are stable enough to act as signaling molecules [35,92]. An increasing body of experimental findings favors the notion that ROS and RNS can act as triggers of dedicated adaptive cellular machinery that increase the organism’s stress resistance [67,93,94]. This includes antioxidant and heat shock responses, cell cycle regulation and apoptosis, DNA repair, fatty acid deacylation-reacylation, unfolded protein responses and autophagy stimulation [95] (Figure 2).

### 3.1. Antioxidant and Heat Shock Responses

Ample evidence suggests that an increased cellular ROS concentration induces cellular defense mechanisms involved in ROS detoxification, such as radical-scavenging enzymes and heat shock proteins [79,96,97]. In single eukaryotes like yeast, increased ROS levels lead to the activation of the redox-responsive transcription factors Msn2/4, which promote gene expression of the ROS-detoxification enzymes such as mitochondrial SOD, or heat shock proteins such as Hsp70 [98].

Multiple redox-sensitive heat shock factors exist in higher eukaryotes. For instance, heat shock factor 1 (HSF1) is normally maintained as a cytosolic monomer through its interaction with Hsp90, a constitutively expressed heat shock protein. When the cell is exposed to elevated levels of ROS, there is an accumulation of unfolded proteins that compete with HSF1 for Hsp90 binding. HSF1 released from the complex forms a homotrimer that can translocate into the nucleus and act as a transcription factor to promote target genes including Hsp27 and Hsp70 [99,100].

Apart from these heat shock factors, increased ROS concentration in mammals also results in the activation of additional transcription factors such as nuclear factor kappa B (NFκB) or cyclic AMP response element binding protein (CREB). These transcription factors trigger the expression of antioxidant enzymes, anti-apoptotic protein Bcl-2, and acute-phase proteins (haptoglobin, beta-fibrinogen) [101]. In agreement with the hormesis concept, rodents exposed to CR exhibit elevated antioxidant defense capabilities [97,102–104], as well as enhanced heat shock protein production [97,103]. Furthermore, two different research groups proposed the hypoxia-inducible factor (HIF-1) as a convergence point of ROS hormesis and hypoxic signaling [105,106].

### 3.2. Cell Cycle Regulation and Selective Apoptosis

Multicellular organisms respond to ROS hormesis in a variety of ways that promote the organism’s survival as a whole. For example, cell growth and proliferation is orchestrated by protein kinase signaling pathways. Moderate concentration of ROS can stimulate cell proliferation by shifting the equilibrium between the phosphorylated and dephosphorylated forms of mitogen-activated kinases (MAPK) [107,108]. Specifically, the active site of the tyrosine phosphatase involved in the MAPK pathway contains a cysteine residue that is hypersensitive to ROS [109]. Thus, this phosphatase is easily inactivated under elevated ROS. In turn, this causes a shift of balance towards the phosphorylated (active) receptor kinase molecules, resulting in an increased kinase activity. The net result is essentially the same as MAPK activation by growth factors [110,111].

On the other hand, when the dramatic ROS damage leads to extensive mitochondrial membrane permeabilization, the alternative strategy of the organism is to activate programmed cell death. For instance, increased production of hydrogen peroxide can cause the leakage of cytochrome c into the cytosol. Cytochrome c subsequently complexes with Apaf-1 (apoptosis protein-activating factor 1), dATP, and procaspase-9 to form an apoptosome. Facilitated by oxidative modification, caspase-9 in turn induces caspase-3 and caspase-7 activation [79,112–114]. This ultimately switches on the downstream apoptosis programs [99]. Relatedly, recent studies have pointed out a possible conserved link between the lifespan-modulating effect of p53 (a tumor suppression protein) and its role in the regulation of hydrogen peroxide-mediated cell death [115].

### 3.3. Crosstalk of ROS Hormesis and the Unfolded Protein Response

Unfolded protein responses (UPR) represent another central mechanism of ROS hormesis in CR and aging [13]. Metabolic stress-induced ROS-upregulation can trigger UPR—the endoplasmic reticulum-centered stress responses—in which several transcription regulating proteins are involved. This includes protein kinase RNA-like ER kinase (PERK), activating transcription factor 6 (ATF6) and inositol requiring endoplasmic reticulum RNAse alpha isoform (IRE1) [116].

PERK directly phosphorylates and activates the transcription factor NF-E2-related factor-2 (Nrf2). This contributes to cellular redox homeostasis by inducing the expression of various antioxidant genes and molecular chaperons in the ER [117,118]. ATF6 is cleaved by site-1 protease (S1P) and site-2 protease (S2P) in the Golgi apparatus to become activated. The cleaved *N*-terminal fragment of ATF6 acts as a transcription factor that migrates to the nucleus, where it induces expression of genes containing the ER stress response element. First described in yeast, IRE1 is susceptible to phosphorylation under elevated ROS. Phosphorylated IRE1 in turn cleaves the XBP1 (X-box binding protein 1) mRNA, resulting in the production of XBP1 proteins. XBP1 is a transcription factor that induces the expression of GRP78 (glucose-regulated protein 78), among other ER-stress proteins [63]. Intrinsically, these three arms of the URP transduction are activated sequentially, with PERK being activated most rapidly, followed by ATF6 and lastly IRE1. Thus, time is allowed for the cell to resolve the stress and promote cell survival. However, if the ROS damages exceed a certain threshold, excessive activation of IRE1 ultimately allows the cell death programs to take over [99]. Activation of the UPR through this process has been observed in post-mortem brain tissues of Parkinson’s disease and Alzheimer’s disease patients [119].

### 3.4. Autophagy Stimulation

Autophagy is a cellular catabolic process that is believed to act against aging, neurodegeneration and cancer [120,121]. The autophagy process involves a myriad of genes that orchestrate the selective engulfment of cytosol and cell organelles, autophagosome formation, and the trafficking and fusion of the autophagosome with the lysosome for final degradation. The autophagosome formation is initiated by Beclin 1 in mammals (Atg6 in yeast). Maturing of the autophagosome and its fusion with the lysosome requires LC3, one of the mammalian homologues of Atg8 in yeast [122]. Specifically, one of the autophagy genes, Atg4, is a cystein protease hyper-sensitive to ROS. At this juncture, ROS and RNS can act as a molecular switch via the reactive thiol groups. Upon elevated ROS in the cell, Atg4 becomes predominantly oxidized. This in turn causes the accumulation of Atg8 phosphoethanolamine precursor that is required for the autophagosome formation [123]. The stimulation of autophagy leads to increased turnover of proteins and defective mitochondria via lysosomal pathways [120]. In addition, by sequestering cytochrome c, autophagy may also delay or prevent apoptosis, thus providing an opportunity for cellular recovery [79].

Autophagy up-regulation has been implemented in lifespan extensions induced by CR and under administration of CR-mimetics such as sirtuin activators [124], rapamycin [125], resveratrol [85,120] and spermidine [126,127]. Intrinsically, autophagy-based selective cellular partial degradation is essential for post-mitotic tissues like the brain. Matus and coworkers have proposed autophagy as a major protective mechanism underlying ROS hormesis to overcome neurodegeneration [79]. This so-called “neurohormetic” effect of autophagy has been shown to suppress disease progression in animal models of Alzheimer’s disease, Parkinson’s disease, and stroke [128].

## 4. Possible Additive Nature of ROS Hormesis

Taken together, a large body of evidences supports the notion that several longevity-promoting interventions including CR and CR-mimetics (as well as physical exercise) may converge by causing an activation of mitochondrial oxygen consumption to promote increased formation of ROS. These ROS serve as a hormetic agent to activate a variety of downstream adaptive responses, which culminate in increased stress resistance, neuronal protection, and longevity [57,129].

Given the forthcoming consensus regarding the possible beneficial effects of ROS hormesis, and keeping in mind their obvious highly damaging potential, two open questions arise: First, what is the mechanistic foundation of the biphasic dose response dynamic? Second and more importantly, what are the major determinants of the hormetic window? These two questions led us to evaluate the quantitative data in the literature.

**Figure 3:**
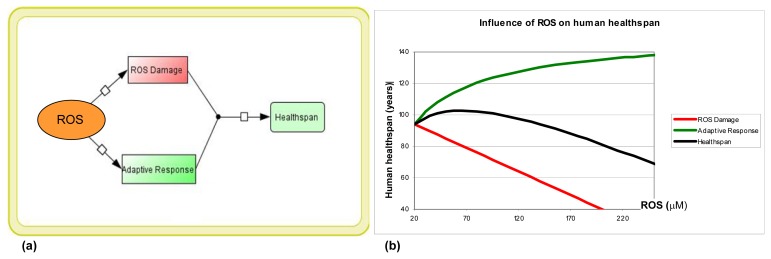
ROS hormesis was modeled by an integration of ROS-induced damage and ROS-induced adaptive responses with different kinetic parameters. (**a**) Model construction employing the software CellDesigner (www.celldesigner.org); (**b**) Simulation results of the model. Red curve: ROS-induced macromolecular damage is negatively and linearly correlated with healthspan. Green curve: ROS-induced adaptive responses are positively correlated with healthspan, bearing a threshold-saturation kinetic (modeled here with Michaelis-Menten kinetics). Black curve: the addition of red and green curve values gave the biphasic hormetic dose-curve with an optimal ROS dose at around 70 μM, which is correlated with maximized human healthspan.

From a study of Bensaad and co-workers on the complex interplay of intracellular ROS and the p53-inducible proteins, it could be deduced that ROS-induced damage may be considered as linearly correlated with ROS concentration [130], whereas ROS-induced adaptive responses, such as DNA repair or autophagy stimulation, supposedly take a substrate-saturation kinetic. Based on these data, we set out to formulate a mathematical model attempting to describe the bifurcated effects of ROS. In our conceptual model, the correlation of human healthspan with ROS concentration was modeled by an additive effect of two elemental reactions: (i) ROS-induced damage; and (ii) ROS-induced adaptive responses. In the first elementary reaction, the ROS-induced damage was negatively, and linearly correlated with the ROS concentration. In the second reaction, the ROS-induced adaptive responses were set to be positively correlated with ROS, but bearing a saturation curve of ROS at about 200 μM (Figure 3a) [130]. This dynamic property was modeled with Michaelis-Menten kinetics, which is frequently used as an approximation of the substance saturation phenomenon.

Combined, our model simulation gave a bi-phasic dose-response curve regarding ROS concentration vs. healthspan, which is typical for the hormesis phenomenon (Figure 3b). No significant effect was seen at ROS levels less than 20 μM. However, a beneficial or healthspan-extending effect of ROS was demonstrated with increasing ROS concentration, with an optimal ROS concentration (hormetic window) lying around 70 μM. ROS concentration higher than 120 μM was associated with a shortened healthspan. Thus, based on our model setting, ROS-induced hormesis in aging could be considered an integration of two opposite effects of ROS: ROS-induced damage, which is expressed as a linear descending curve in the ROS *vs.* damage diagram, and the ROS-stimulation of adaptive responses. This model presents a novel computational insight into the bi-phasic curve of ROS hormesis. Interestingly, in a study on human keratinocyte aging, 60 μM of hydrogen peroxide was shown to exert beneficial hormetic effects on telomere length maintenance [131]. This is in keeping with our modeling prediction.

Notably, based on our mathematical model, the optimal hormetic dose of ROS for maximal healthspan is dependent on the individual kinetic parameters chosen for both ROS-induced damage and ROS-induced adaptive responses. Accordingly, maximizing the benefit of hormesis requires an optimized ROS concentration that enhances the stress response, while still staying far away from possible detrimental toxicity of ROS.

## 5. Potential Therapeutic Value of ROS Hormesis

Having these solid hormesis effects in hand, it is particularly inviting to think of its therapeutic potential. Oxidative stress and mitochondrial dysfunction have been implicated in multiple age-related pathologies [40]. Regarding neurodegenerative diseases, imbalance of cellular homeostasis results in protein misfolding and accumulation of insoluble protein fibrils and aggregates (both inside and outside the cell) [132]. With age, this further impedes the brain function accompanied by proteostasis breakdown. It is therefore tempting to consider interventions that employ transient ROS elevation to stimulate numerous endogenous cellular defense mechanisms.

Indeed, several experimental trials of ROS hormesis as a potential therapeutic strategy against ND and other age-related diseases have already been reported. For instance, low dose radiation, a process that generate ROS (albeit extracellular), has been shown to exert neuroprotective effects in mouse models of *retinitis pigmentosa*, a hereditary, progressive neurodegeneration that ultimately leads to blindness [133]. Furthermore, in pancreatic beta-cells, sublethal exogenous H_2_O_2_ has been shown to induce secondary repair and defense mechanisms that counteract diabetes [134]. In line with this, low doses of nicotine have been shown to stimulate mitochondrial autophagy in cultured human endothelial cells, possibly via a transient increase of ROS production [135,136]. Whether this could be associated with the beneficial effect on Parkinson’s disease prevention remains to be elucidated.

## 6. Conclusions and Perspectives

All living systems have the intrinsic ability to respond and adapt to external and internal sources of disturbance [57]. Although the hazardous damaging effect of ROS is a settled issue, data summarized here support the theory that hormetic effects of ROS in aging and ND are obvious. In this regard, caloric restriction, CR-mimetics and physical exercise probably share several common mechanistic features that are mediated by increased ROS levels due to enhanced mitochondrial activity. This subsequently induces the organism’s adaptive responses and ultimately results in lifespan-extension and health promotion [67].

We welcome the renaissance of hormesis, but at the same time suggest that the word hormesis might currently be overblown, probably due to the great anticipation of the therapeutic potential of this phenomenon. It needs to be reiterated here that, all in all, hormetic effects tend to be small in size relative to the potentially fatal effects of these toxins. A better understanding of the biology underlying the bi-phasic dose response would be warranted to finally utilize these strategies for therapy promoting longer healthspan. Here, a critical key to the success of future medical intervention using the principle of hormesis is to get the right dose—the hormetic window [137], which will be anything but trivial [28,138]. This is because the parameters of each underlying sub-reaction are all dependent on individual reaction mechanisms. The dimension of the homeodynamic space of each patient is determined by their interacting genetic and epigenetic networks, which are part of the uniqueness of the individual [31,139]. Consequently, future investigations need to pay special attention to the inter-individual quantitative feature of the hormetic dose response when exploring therapy susceptibility [140]. This necessitates the systematic collection of descriptive datasets on cohort study that facilitate sophisticated theoretical approaches [141]. Albeit tedious, this would be an obligatory measure in ultimately utilizing the therapeutic potential of ROS hormesis against aging and age-related degenerative diseases.

In his later years, Friedrich Nietzsche seems to have given up being “stronger”, instead trying to avoid harshness and end his life at ease (he died at the age of 56). On the contrary, however, Mr. Helmut Schmidt still consumes a large number of cigarettes at every occasion. Perhaps his wisdom has led him to his personal optimal hormesis dose? Let’s keep our fingers crossed.
